# Clinicopathologic characteristics and outcomes of papillary thyroid carcinoma in younger patients

**DOI:** 10.1097/MD.0000000000019795

**Published:** 2020-04-10

**Authors:** Yi Lu, Lin Jiang, Chao Chen, Haitao Chen, Qinghua Yao

**Affiliations:** a Department of Nutrition, Institute of cancer research and basic medical sciences of Chinese Academy of Sciences, Cancer hospital of University of Chinese Academy of Sciences, Zhejiang Cancer Hospital; b Department of Head and Neck Surgery, Institute of cancer research and basic medical sciences of Chinese Academy of Sciences, Cancer hospital of University of Chinese Academy of Sciences, Zhejiang Cancer Hospital; c First Clinical College of Zhejiang Chinese Medical University; d Department of Integrated Chinese and Western Medicine, Institute of cancer research and basic medical sciences of Chinese Academy of Sciences, Cancer hospital of University of Chinese Academy of Sciences, Zhejiang Cancer Hospital; e Key Laboratory of Traditional Chinese and Western Medicine Oncology of Zhejiang Province, Hangzhou, P.R. China.

**Keywords:** clinicopathologic features, disease-free survival, papillary thyroid carcinoma, recurrence

## Abstract

In the 7th edition of AJCC staging system, cervical lymph node metastases (LNM) in papillary thyroid carcinoma (PTC) is considered as a poorer prognostic indicator only in patients aged 45 years or older, but as a low-risk factor in patients younger than 45 years. The objective of this study is to investigate the influence of cervical LNM on prognostic outcomes of young patients (<45 years’ old) with PTC.

We carried out a retrospective analysis of 1896 PTC patients younger than 45 years’ old at diagnosis, who were firstly treated in our department between January 2005 and December 2014. Clinicopathologic features, recurrences, disease-free survival (DFS) were recorded and analyzed.

A total of 1896 consecutive patients were identified, comprising of 426 males and 1470 females after a median follow-up period of 40 months (3–129 months) from initial surgery to disease recurrence or to the end of follow-up. The rate of recurrence was 2.16% (n = 41). The DFS rates for a 1-year, 3-year, or 5-year team were 99.1%, 97.8%, or 97.4%, respectively. Univariate analysis showed that diagnosed age ≤30 years, tumor size >1.0 cm, extrathyroidal extension, multifocal lesions, lesions in bilateral lobes, central neck LNM, and lateral neck LNM were associated with a worse DFS. Multivariate analysis showed that only central neck LNM and lateral neck LNM were significant independent prognostic factors for DFS (*P* < .001). For patients with papillary thyroid microcarcinoma, cervical LNM were also identified as independent risk factors for DFS (*P* < .001).

LNM have prognostic significance for DFS in PTC patients younger than 45 years. It indicated that PTC patients (<45 years old) with LNM, especially lateral neck LNM, were understaged by the 7th edition of AJCC staging system. Thus, radical resection of primary tumor and metastatic lymph nodes, frequent follow-up, and strict TSH suppression should be taken for young patients with PTC.

## 1 Introduction

Thyroid cancer is a common malignant tumor of the head and neck, and the most common sort of endocrine tumors, accounting for >90% of all endocrine malignancies.^[1]^ Also, thyroid cancer is the most rapidly growing cancer in last 2 decades in the world. Among all types of thyroid cancers, papillary carcinoma has the best prognosis. The age at diagnosis is one of the most important factors influencing its staging and the following treatments. In the 7th edition of AJCC staging system, patients of differentiated thyroid carcinomas younger than 45 years were divided into 2 stages: Stage I for those without distant metastases, and Stage II for those with distant metastases. The disease-free survival (DFS) rate is higher for patients younger than 45 years compared to that of the patients older than 45 years, and the mortality rate is low. However, even been clarified into the same stage, different T grade, different N status usually indicate different outcomes, especially the recurrence and DFS rate. Our research discussed the relationship between clinicopathologic features and the outcomes of papillary thyroid carcinoma (PTC) patients younger than 45 years.

## 2 Materials and methods

### 2.1 Patients

We studied 1896 consecutively treated patients who were firstly diagnosed as PTC via the histopathologic examination in the Department of Head and Neck Surgery, Zhejiang Cancer Hospital, China, between January 2005 and December 2014. All patients enrolled were younger than 45 years at diagnosis, and underwent surgical treatment. We compared clinicopathologic features, recurrence rates, DFS rates between different groups. The following variables were considered: age, sex, extrathyroidal extension, multifocality, and lymph node metastases (LNM).

### 2.2 Surgery

All the patients were treated by radical surgery, either hemithyroidectomy, or total thyroidectomy. Central neck dissection was performed routinely, whereas lateral neck dissection was performed only if the metastases were proven by fine-needle aspiration biopsy (FNAB) or highly suspected by radiological examination.

### 2.3 Follow-up

After surgery, all the patients took TSH suppression therapy, and followed by physical examination, laboratory and ultrasound examination every 3 months for the first year, every 6 months the following 3 years, and every 1 year thereafter. Ultrasonography-guided FNAB was performed when there was clinical suspicion of LNM or recurrence in the remnant thyroid. Once the recurrences had been confirmed, the follow-up ended, and patients underwent further treatments.

### 2.4 Ethics statement

This study was performed in compliance with the Declaration of Helsinki, approved by an independent ethics committee/institutional review board at Cancer hospital of University of Chinese Academy of Sciences (Zhejiang Cancer Hospital), and conducted in accordance with the approved guidelines. Data released from our database do not require informed patient consent because they contain no identifiers and are publicly available.

### 2.5 Statistical analysis

We used *χ* ^2^ test to examine the relationship between clinicopathologic characteristics and the recurrences. The Kaplan–Meier method and the log rank test were used to estimate and compare the DFS between different groups. Multivariate analysis was performed by Cox-Hazard regression model. Results were considered statistically significant when *P* < .05. Statistical analyses were performed by SPSS software ver. 19.0 for Windows.

## 3 Results

### 3.1 Clinicopathologic features

A total of 1896 consecutive patients were identified in our research, comprising of 426 males and 1470 females. The median age at diagnosis was 35.7 (±6.5) years old. The median tumor diameter was 1.02 cm (0.1–8.0 cm). In addition, 143 (7.5%) patients had extrathyroidal extension, 149 (7.9%) patients had multifocal lesions, 290 (15.3%) patients had bilateral lobe lesions, 921 (48.6%) patients had central neck LNM, and 231 (12.2%) patients had lateral neck LNM (Table 1).

**Table 1:**
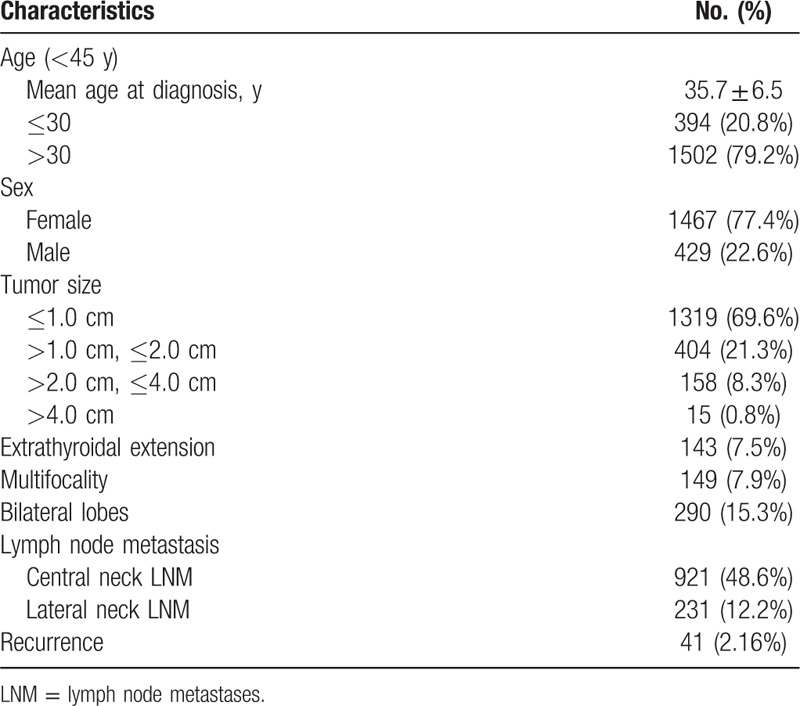
Demographic and clinicopathological characteristics of all the 1896 patients.

### 3.2 Recurrences

The median follow-up period from initial surgery to disease recurrence or to the end of follow-up was 40 months (3–129 months). The rate of recurrence was 2.16% (n = 41, Table 2), including local recurrence and regional LNM. The following factors, including age at diagnosis ≤30 years old, tumor size >1.0 cm, extrathyroidal extension, multifocality, bilateral lobe lesions, central neck LNM, and lateral neck LNM, were significantly associated with recurrences (*P* < .05). However, the recurrence rate between males and females had no significant difference (*P* = .63, Table 2).

**Table 2:**
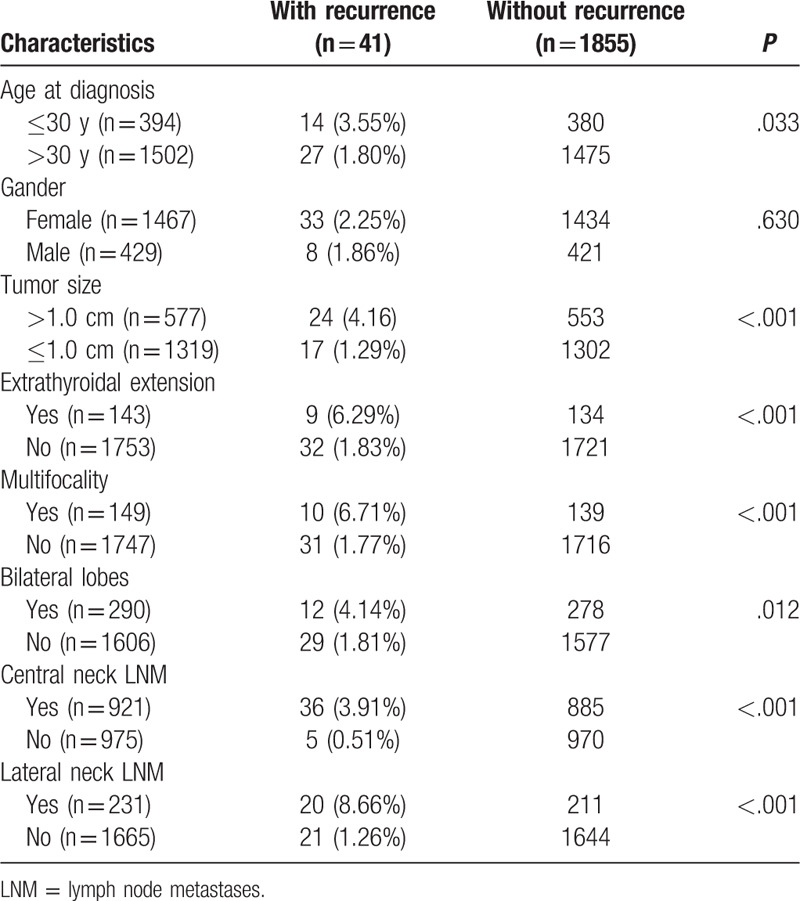
Relationship between clinicopathological characteristics and recurrence.

### 3.3 DFS

**Figure 1:**
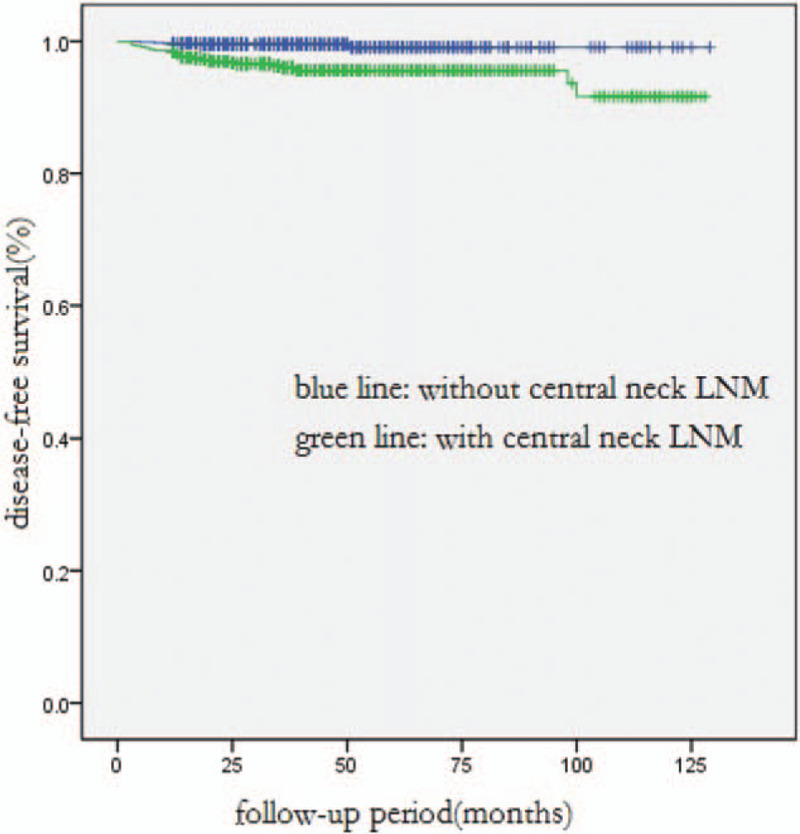
Comparison of disease-free survival between all patients with and without central neck LNM (*P* < .001).LNM =

**Figure 2:**
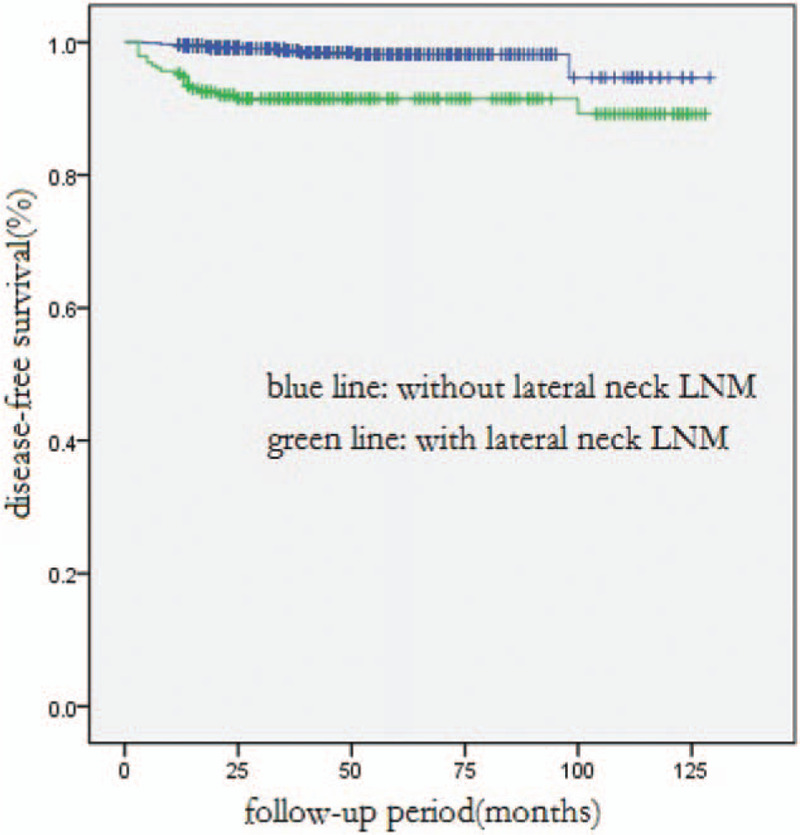
Comparison of disease-free survival between all patients with and without lateral neck LNM (*P* < .001).

Of all the patients, the DFS rate for a 1-year, 3-year, or 5-year term was 99.1%, 97.8%, or 97.4%, respectively. In univariate analysis, DFS was significantly associated with younger age at diagnosis (*P* = .033), larger tumor size (*P* = .001), extrathyroidal extension (*P* = .002), multifocal lesions (*P* < .001), lesions in bilateral lobes (*P* = .007), and LNM (*P* < .001). Multivariate analysis showed that only central neck LNM and lateral neck LNM were independent predictors for shorter DFS (*P* = .010, *P* = .001, respectively, Table 3, Figs. 1 and 2).

**Table 3:**
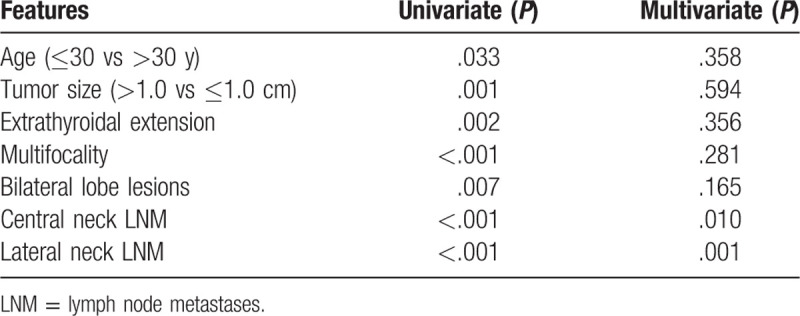
Univariate and multivariate analyses of DFS for all patients.

**Figure 3:**
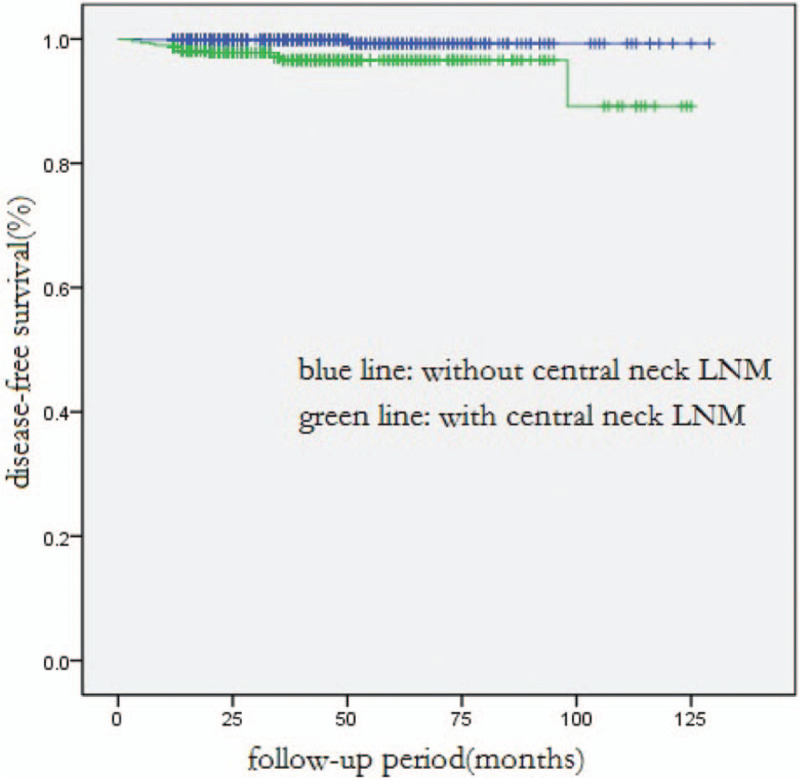
Comparison of disease-free survival between PTMC patients with and without central neck LNM (*P* < .001).

**Figure 4:**
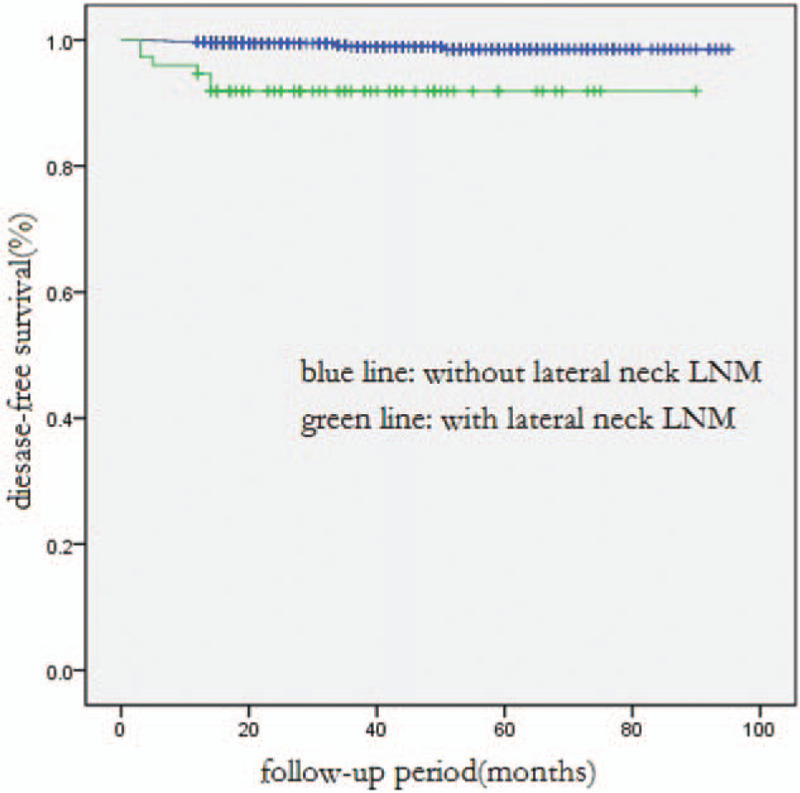
Comparison of disease-free survival between PTMC patients with and without lateral neck LNM (*P* < .001).

Next, we conducted a subgroup analysis on patients with PTMC (papillary thyroid microcarcinoma (PTMC), defined as tumor size equal to or less than 1 cm), and we discovered that patients with central and lateral neck LNM were more likely to recur than patients without. In multivariate analysis, central neck LNM and lateral neck LNM were also independent risk factors for shorter DFS in PTMC patients (Table 4, Figs. 3 and 4).

**Table 4:**
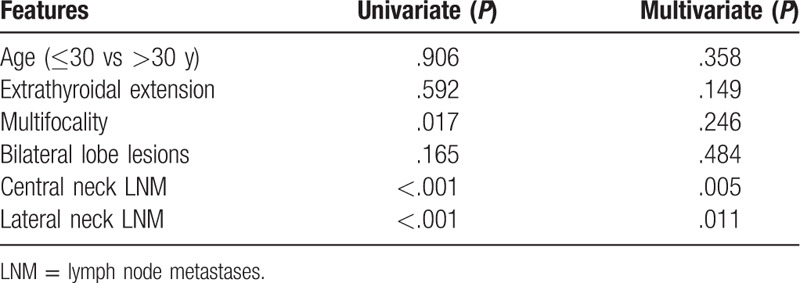
Univariate and multivariate analyses of DFS for PTMC patients.

## 4 Discussion

Papillary thyroid cancer is the fastest increasing malignancy in the world. The prognosis is generally excellent with appropriate therapy. Patients younger than 45 years are considered at low risk and have a favorable prognosis, even in combination with locoregional advanced disorders. In the 7th edition of American Joint Committee on Cancer (AJCC) staging system, patients younger than 45 years with differentiated thyroid cancer were classified as only 2 stages, Stage I for those without distant metastases, and Stage II for those with distant metastases. The status of cervical lymph nodes has not gotten particular attention.

Recent studies reported that the rate of central neck LNM in PTC ranged from 30% to 90%,^2^,^3^,^4^ and the rate of lateral neck LNM ranged from 20% to 70%.^5^,^6^,^7^ In our research, the central neck LNM rate was 48.6%, which was consistent with previous reports. According to the Chinese Association of Thyroid Oncology guideline for PTMCs, prophylactic central lymph node dissection (CLND) was performed routinely in our department which identified a significant percentage of patients with LNM. In the current ATA guideline, prophylactic CLND was not recommended for patients with clinically node-negative (cN0) PTMC. However, some authors argued its application in cN0 PTMC because of the high prevalence of CLNM, although CLNM was not significantly associated with the overall survival according to the public data. In our study, we demonstrated that cervical LNM were associated with shorter DFS in patients younger than 45 years.

The rate of lateral neck LNM was 12.2% in our study, which was lower than the rate reported in previous papers, likely because most patients in our study had PTMCs. Although the application of prophylactic central lymph node dissection (CLND) remains controversial,^8^,^9^,^10^ we perform CLND routinely in our department. Prophylactic lateral lymph node dissection (LLND) was not performed if there was no evidence of metastases based on physical and imaging examination. If there was clinical suspicion of lateral neck LNM, ultrasonography-guided FNAB should be done for confirmation, and then the patient underwent therapeutic LLND. Usually, the LLND included Level II to Level V. Dissection of Level I might be unnecessary as the metastatic rate was rare.^[11]^

Previous studies showed that the mean 10-year survival rate of PTC was >90%.^12^,^13^ The prognosis in young patients was better. The TNM classification established by the American Joint Commission on Cancer (AJCC) showed that, for patients younger than 45 years, there were just 2 stages, Stage I for those without distant metastases, and Stage II for those with distant metastases. However, many patients experienced locoregional recurrences (LRs) and underwent repeated surgeries. The LR rates varied from 2% to 45% in patients with different TNM stages.^14^,^15^,^16^,^17^ In our study, the recurrence rate was 2.16%, lower than the average level, since most of patients in our study were diagnosed at early stage. The risk factors of LR including tumor size >1.0 cm, extrathyroidal extension, multifocality, bilateral lobe lesions, central neck LNM, and lateral neck LNM, were consistent with previous studies^18^,^19^ In our research, patients younger than 30 years bear higher recurrence rate compared to patients between 30 and 45 years of age, indicated that younger patients were more prone to relapse.

In this study, the mortality rate was 0.2%. There were only 4 confirmed deaths, since most were patients with PTMCs and the mean follow-up time was just 40 months. The DFS rate for a 1-year, 3-year, or 5-year team was 99.1%, 97.8%, or 97.4%, respectively. In multivariate analysis of all patients, central neck LNM (*P* = 0.010) and lateral neck LNM (*P* = 0.001) were identified as significant risk factors affecting DFS, especially the lateral neck LNM. The average period length from initial surgery to the first recurrence in patients with and without lateral neck LNM was 14.5 months and 24.5 months, respectively. As reported by other authors, lateral neck LNM was an independent predictive factor of recurrence and disease-free survival.^20^,^21^,^22^,^23^ Moreover, in multivariate analysis of PTMC patients, central neck LNM (*P* = 0.005) and lateral neck LNM (*P* = 0.011) were also significant risk factors of DFS.

## 5 Conclusion

In this study, we demonstrated the prognostic significance of LNM on DFS in PTC patients younger than 45 years. Our study showed that patients with PTC did not always indicate a good prognosis, which was contradictory with previous studies. The presence and absence of metastatic cervical lymph node status led to different outcomes in PTC patients. Those who had positive cervical lymph nodes were more prone to experience recurrence and underwent repeated treatments, which brought extra financial burden and led to a decrease in quality of life. Moreover, patients with cervical LNM had shorter DFS than those without, and might bear the higher mortality rate.

Thus, radical resection of primary tumor and metastatic lymph nodes, frequent follow-up, and strict TSH suppression should be taken for young patients with PTC.

## Author contributions

**Conceptualization:** Qinghua Yao.

**Investigation:** Yi Lu.

**Methodology:** Lin Jiang.

**Project administration:** Chao Chen.

**Software:** Lin Jiang, Haitao Chen.

**Writing – original draft:** Yi Lu.

**Writing – review & editing:** Qinghua Yao.

## References

[1] Siegel RL Miller KD Jemal A Cancer Statistics, 2017. CA Cancer J Clin 2017;67:7–30. 28055103 10.3322/caac.21387

[2] Sun W Lan XB Zhang H Risk factors for central lymph node metastasis in cN0 papillary thyroid carcinoma: a systematic review and meta-analysis. PLoS One 2015;10:e0139021. 26431346 10.1371/journal.pone.0139021 PMC4592212

[3] Qu H Sun GR Liu Y Clinical Risk factors for central lymph node metastasis in papillary thyroid carcinoma: A systematic review and meta-analysis. Clin Endocrinol 2015;83:124–32. 10.1111/cen.12583 25130203

[4] Ma B Wang Y Yang SW Predictive factors for central lymph node metastasis in patients with cN0 papillary thyroid carcinoma: a systematic review and meta-analysis. Int J Surg 2016;28:153–61. 26944586 10.1016/j.ijsu.2016.02.093

[5] Mulla MG Knoefel WT Gilbert J Lateral cervical lymph node metastases in papillary thyroid cancer: a systematic review of imaging-guided and prophylactic removal of the lateral compartment. Clin Endocrinol 2012;77:126–31. 10.1111/j.1365-2265.2012.04336.x 22233478

[6] Eskander A Merdad M Freeman JL Pattern of spread to the lateral neck in metastatic well-differentiated thyroid cancer: A systematic review and meta-analysis. Thyroid 2013;23:583–92. 23148663 10.1089/thy.2012.0493

[7] Yuce I Cagli S Bayram A Regional metastatic pattern of papillary thyroid carcinoma. Eur Arch Otorhinolaryngol 2010;267:437–41. 19585137 10.1007/s00405-009-1032-6

[8] Lang BH Ng SH Lau LL A systematic review and meta-analysis of prophylactic central neck dissection on short-term locoregional recurrence in papillary thyroid carcinoma after total thyroidectomy. Thyroid 2013;23:1087–98. 23402640 10.1089/thy.2012.0608

[9] Conzo G Docimo G Mauriello C The current status of lymph node dissection in the treatment of papillary thyroid cancer. A literature review. Clin Ter 2013;164:e343–6. 24045534 10.7417/CT.2013.1599

[10] Chen Q Zou XH Wei T Prediction of ipsilateral and contralateral central lymph node metastasis in unilateral papillary thyroid carcinoma: a retrospective study. Gland Surg 2015;4:288–94. 26312214 10.3978/j.issn.2227-684X.2015.05.06 PMC4523630

[11] Kumar S Burgess C Moorthy R The extent of lateral lymph node dissection in differentiated thyroid cancer in the N+ neck. Eur Arch Otorhinolaryngol 2013;270:2947–52. 23519682 10.1007/s00405-013-2434-z

[12] Pelizzo MR Merante BI Toniato A Diagnosis, treatment, prognostic factors and long-term outcome in papillary thyroid carcinoma. Minerva Endocrinol 2008;33:359–79. 18923371

[13] Matsuzu K Sugino K Nagahama M Thyroid lobectomy for papillary thyroid cancer: long-term follow-up study of 1,088 cases. World J Surg 2014;38:68–79. 24081532 10.1007/s00268-013-2224-1

[14] Said M Fujimoto M Franken C Preferential use of total thyroidectomy without prophylactic central lymph node dissection for early-stage papillary thyroid cancer: oncologic outcomes in an integrated health plan. Perm J 2016;20:22–6. 10.7812/TPP/15-251 PMC5101086 27723445

[15] Zhu J Wang XL Zhang XX Clinicopathologic features of recurrent papillary thyroid cancer. Diagn Pathol 2015;10:96–8. 26168921 10.1186/s13000-015-0346-5 PMC4501206

[16] Joo JY Jin J Seo ST Recurrence in regional lymph nodes after total thyroidectomy and neck dissection in patients with papillary thyroid cancer. Oral Oncol 2015;51:164–9. 25435434 10.1016/j.oraloncology.2014.11.004

[17] Ito Y Kudo T Takamura Y Lymph node recurrence in patients with N1b papillary thyroid carcinoma who underwent unilateral therapeutic modified radical neck dissection. World J Surg 2012;36:593–7. 22207493 10.1007/s00268-011-1391-1

[18] Suh YJ Kwon H Kim SJ Factors affecting the locoregional recurrence of conventional papillary thyroid carcinoma after surgery: a retrospective analysis of 3381 patients. Ann Surg Oncol 2015;22:3543–9. 25743326 10.1245/s10434-015-4448-9

[19] Girardi FM Barra MB Zettler CG Variants of papillary thyroid carcinoma: association with histopathological prognostic factors. Braz J Otorhinolarygol 2013;79:738–44. 10.5935/1808-8694.20130135 PMC9442390 24474487

[20] Chereau N Buffet C Tresallet C Recurrence of papillary thyroid carcinoma with lateral cervical node metastases: predictive factors and operative management. Surgery 2016;159:755–62. 26435440 10.1016/j.surg.2015.08.033

[21] Liu FH Kuo SF Hsueh C Postoperative recurrence of papillary thyroid carcinoma with lymph node metastasis. J Surg Oncol 2015;112:149–54. 26175314 10.1002/jso.23967 PMC5034820

[22] Schneider DF Chen H Sippel RS Impact of lymph node ratio on survival in papillary thyroid cancer. Ann Surg Oncol 2013;20:1906–11. 23263904 10.1245/s10434-012-2802-8 PMC3609925

[23] Meer SG Dauwan M Keizer B Not the number but the location of lymph nodes matters for recurrence rate and disease-free survival in patients with differentiated thyroid cancer. World J Surg 2012;36:1262–7. 22270993 10.1007/s00268-012-1427-1 PMC3348473

